# How cells kill a "killer" messenger

**DOI:** 10.7554/eLife.16076

**Published:** 2016-04-27

**Authors:** Cosmin Saveanu, Alain Jacquier

**Affiliations:** 1 Institut Pasteur, Paris, France; 1 Institut Pasteur, Paris, Francealain.jacquier@pasteur.fr; 2 CNRS UMR3525, Paris, France; 2 CNRS UMR3525, Paris, France

**Keywords:** mRNA decay, NMD, Gadd45, Drosophila, Upf1, Upf2, *D. melanogaster*

## Abstract

Establishing a link between the nonsense-mediated decay pathway and a gene associated with programmed cell death could explain why this pathway is essential in most, but not all, eukaryotes.

**Related research article** Nelson JO, Moore KA, Chapin A, Hollien J, Metzstein MM. 2016. Degradation of Gadd45 mRNA by nonsense-mediated decay is essential for viability. *eLife* **5**:e12876. doi: 10.7554/eLife.12876

**Image** Cell death caused by defects in nonsense-mediated decay in fruit flies (left) can be prevented if the flies also lack the GADD45 protein (right)

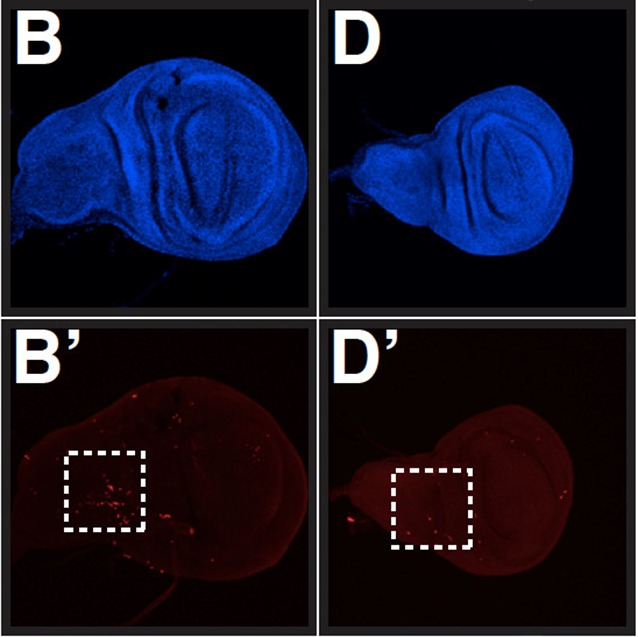

All messenger RNA (mRNA) transcripts contain a stop codon and the ribosome – the molecular machine that translates the transcript to make a protein – stops when it reaches this codon. The stability of mRNAs, as for all types of RNA molecules, can be compromised by the presence of stop codons in positions that are “inappropriate”. Nonsense-mediated mRNA decay is the pathway that degrades such transcripts (Lykke-Andersen and Jensen, 2015; Kervestin and Jacobson, 2012). The mechanisms that allow a "normal" stop codon to be distinguished from a stop codon that induces nonsense-mediated decay are still unclear: however, RNA molecules that are sensitive to nonsense-mediated decay are in general characterized by a short coding sequence followed by a long untranslated region (Figure 1A).

**Figure 1.**
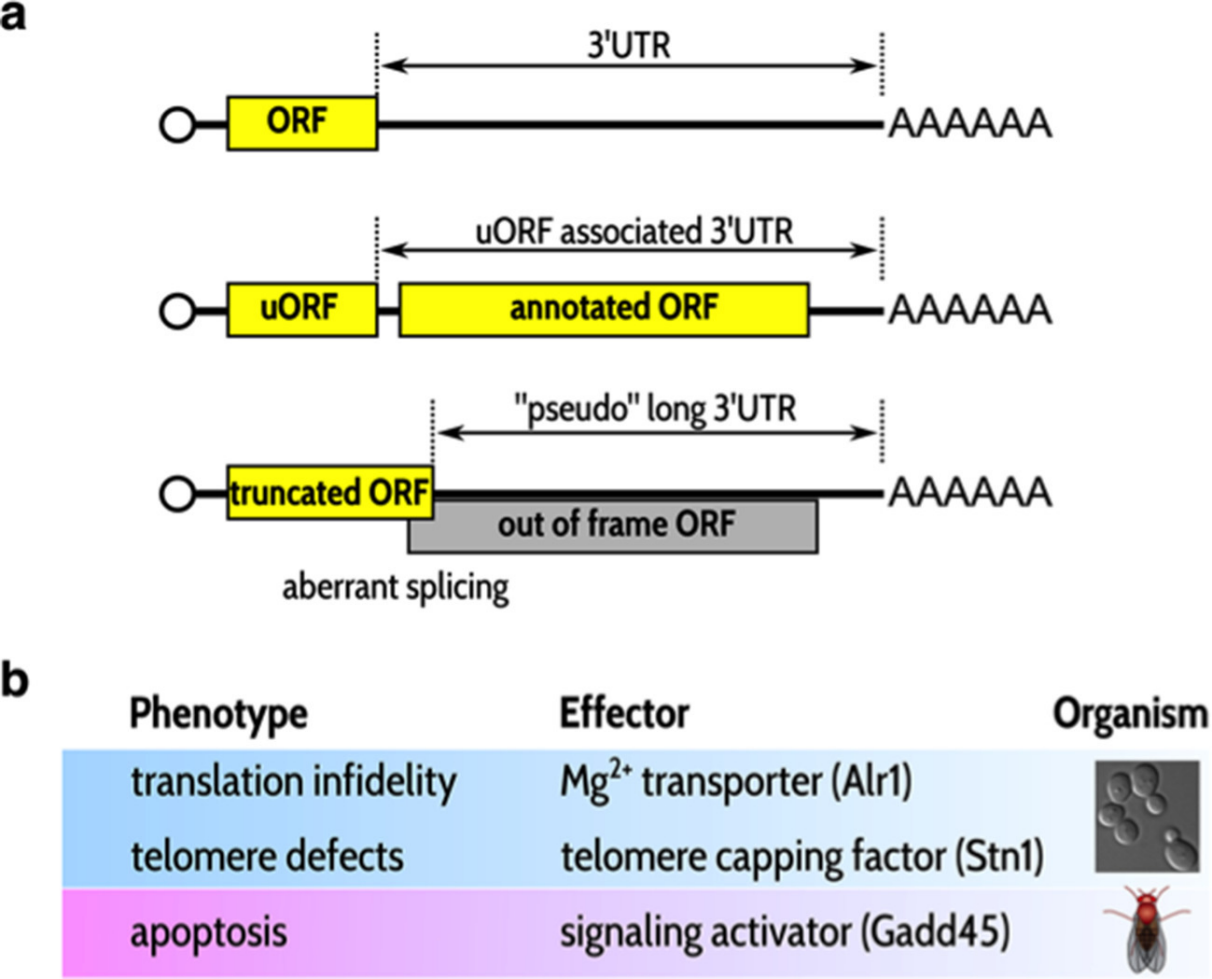
Typical substrates for nonsense-mediated decay (NMD) and examples of mRNAs whose stabilization can lead to specific phenotypes in the absence of NMD. (A) NMD substrates typically have short open reading frames (ORFs) followed by long 3’ untranslated regions (3’ UTRs; top). Some NMD substrates also have an annotated ORF (middle). Short ORFs followed by relatively long 3'UTRs are also randomly present in incorrectly spliced mRNA transcripts (bottom). (**B**) Three examples of phenotypic traits observed in the absence of NMD; each trait is mediated by the stabilization of the mRNA shown in the second column. The first two links were observed in yeast (Johansson and Jacobson, 2010; Addinall et al., 2011); the last was observed by Nelson et al. in flies.

Inactivating the nonsense-mediated decay (NMD) pathway prevents the development of mice, zebrafish, fruit flies and plants, but the roundworm *C. elegans* and two species of yeast (*S. cerevisiae* and *S. pombe*) can survive reasonably well without it. Since this pathway affects thousands of different transcripts, it is not clear what makes it essential for the survival of so many species. Are its effects important for only one or very few transcripts, or is it important because of the cumulative degradation of a large number of potentially harmful mRNA transcripts? Now, in eLife, Mark Metzstein and colleagues at the University of Utah – including Jonathan Nelson as first author *–* present new evidence that strengthens the hypothesis that the lethality caused by inactivating the NMD pathway results from the stabilization of a small number of mRNA transcripts (Nelson et al., 2016).

Nelson et al. used the power of *Drosophila* genetics to screen for mutations that increased the ability of fruit fly embryos to survive the partial inactivation of the NMD pathway. One of the mutations that most improved the ability of the flies to survive was a mutation that inactivated a gene called GADD45, which promotes a process of programmed cell death called apoptosis.

Three observations were critical in focusing the researchers' attention on GADD45. First, the Gadd45 protein is a signaling molecule that can induce the apoptosis of eukaryotic cells (as well as performing other functions; see review by Tamura et al. (2012). Coincidentally, no versions of Gadd45 have been detected in the organisms in which the NMD pathway is not essential for survival (Kriventseva et al., 2015). Second, the Gadd45 mRNA is in the 'top 10' of the transcripts that are strongly stabilized in the absence of nonsense-mediated decay in *Drosophila* larvae, as shown previously by the Metzstein laboratory (Chapin et al., 2014). Third, the genetic screen performed by Nelson et al. also showed that inactivating a protein kinase called Mekk1 – which is the best-known target of Gadd45 – could also improve the survival of the fruit fly embryos.

Together these data suggest that, in *Drosophila*, the increase in the levels of GADD45 mRNA transcripts that occurs when nonsense-mediated decay is inactivated can send a signal to trigger apoptosis. Degrading these transcripts effectively prevents apoptosis; hence, nonsense-mediated decay can control a 'killer' messenger.

One of the most impressive outcomes of the *Drosophila* genetic screen was that its results could be partially recreated in mammalian cells. Inactivating nonsense-mediated decay reduced the viability of cultured mouse and human cells. However, Nelson et al. found that this loss of viability could be partially reversed by inactivating GADD45B, which is one of the three genes that code for GADD45 proteins in mammals (Tamura et al., 2012). The partial nature of this reversal might be explained by the fact that mammals have three genes for GADD45, whereas flies only have one. However, it could also indicate that other targets of the NMD pathway contribute to the loss of viability.

The fact that GADD45 mRNA transcripts are destabilized by nonsense-mediated decay in both *Drosophila* and mammalian cells suggests that RNA instability might be an evolutionarily conserved requirement for the signaling role of the Gadd45 proteins. One can imagine nonsense-mediated decay somehow being a link between the high turnover rate of these transcripts and the translation of these transcripts to produce the Gadd45 protein. For example, a form of stress could temporarily repress the translation process, leading to the accumulation of GADD45 mRNA transcripts. Then, when the cells were relieved of the stress, the accumulated transcripts would produce a burst of Gadd45 proteins that could modulate the ability of the cell to respond to any future stress.

Nelson et al. have established a clear link between a particular phenotype and the stabilization of a specific mRNA transcript in the absence of nonsense-mediated decay in flies, and similar links have been established by other groups working with yeast (Figure 1B). It is more difficult to assign a phenotype to the global destabilization of hundreds or thousands of transcripts by nonsense-mediated decay, as we cannot delete all the corresponding genes to see which phenotype is suppressed. However, the role that such global processes play in the life of cells can still be investigated, albeit in more indirect ways.

For example, working with colleagues, the present authors recently identified a situation in which NMD appears to participate to a genome-wide effect (Malabat et al., 2015). Mapping of transcription start sites in yeast showed that a large variety of RNA molecules are synthesized in parallel with mRNA but are rapidly degraded via nonsense-mediated decay. The regions from which these identified transcripts originate have sequence characteristics biased to favor rapid decay of the aberrant transcripts. That such aberrant transcripts are only tolerated if they become substrates for nonsense-mediated decay is an example of global destabilization being subject to evolutionary selection.

The identification of specific transcripts that use robust nonsense-mediated decay for high turnover, combined with global analyses of targets for the NMD pathway, will improve our understanding of this process and also provide an incentive for researchers to continue investigating both the molecular mechanisms involved in nonsense-mediated decay and the evolution of these mechanisms in eukaryotes.
